# estimateR: an R package to estimate and monitor the effective reproductive number

**DOI:** 10.1186/s12859-023-05428-4

**Published:** 2023-08-11

**Authors:** Jérémie Scire, Jana S. Huisman, Ana Grosu, Daniel C. Angst, Adrian Lison, Jinzhou Li, Marloes H. Maathuis, Sebastian Bonhoeffer, Tanja Stadler

**Affiliations:** 1 Department of Biosystems Science and Engineering, ETH Zurich, Swiss Federal Institute of Technology, Basel, Switzerland; 2 Swiss Institute of Bioinformatics, Lausanne, Switzerland; 3 Department of Environmental Systems Science, ETH Zurich, Swiss Federal Institute of Technology, Zurich, Switzerland; 4 Department of Physics, Massachusetts Institute of Technology, Cambridge, USA; 5 Department of Mathematics, ETH Zurich, Swiss Federal Institute of Technology, Zurich, Switzerland

**Keywords:** R package, Epidemiology, Effective reproductive number, Re, Rt, Surveillance, Monitoring, Outbreak, COVID-19

## Abstract

**Background:**

Accurate estimation of the effective reproductive number ($R_e$) of epidemic outbreaks is of central relevance to public health policy and decision making. We present estimateR, an R package for the estimation of the reproductive number through time from delayed observations of infection events. Such delayed observations include confirmed cases, hospitalizations or deaths. The package implements the methodology of Huisman et al. but modularizes the $R_e$ estimation procedure to allow easy implementation of new alternatives to the currently available methods. Users can tailor their analyses according to their particular use case by choosing among implemented options.

**Results:**

The estimateR R package allows users to estimate the effective reproductive number of an epidemic outbreak based on observed cases, hospitalization, death or any other type of event documenting past infections, in a fast and timely fashion. We validated the implementation with a simulation study: estimateR yielded estimates comparable to alternative publicly available methods while being around two orders of magnitude faster. We then applied estimateR to empirical case-confirmation incidence data for COVID-19 in nine countries and for dengue fever in Brazil; in parallel, estimateR is already being applied (i) to SARS-CoV-2 measurements in wastewater data and (ii) to study influenza transmission based on wastewater and clinical data in other studies. In summary, this R package provides a fast and flexible implementation to estimate the effective reproductive number for various diseases and datasets.

**Conclusions:**

The estimateR R package is a modular and extendable tool designed for outbreak surveillance and retrospective outbreak investigation. It extends the method developed for COVID-19 by Huisman et al. and makes it available for a variety of pathogens, outbreak scenarios, and observation types. Estimates obtained with estimateR can be interpreted directly or used to inform more complex epidemic models (e.g. for forecasting) on the value of $R_e$.

**Supplementary Information:**

The online version contains supplementary material available at 10.1186/s12859-023-05428-4.

## Background

The coronavirus disease 2019 (COVID-19) pandemic has demonstrated that reliable quantification of pathogen transmission is key to an informed and timely public health response during an epidemic [1]. Moreover, accurate knowledge of pathogen transmission is essential for retrospective evaluation of the effectiveness of pharmaceutical and non-pharmaceutical interventions against spreading pathogens [2–4].

The reproductive (or reproduction) number corresponds to the average number of secondary infections caused by an infected individual. The time-varying effective reproductive number $R_\text{e}$ (or $R_t$) is a measure of the pathogen transmission in a population. Several methods have been proposed for its calculation, including those that monitor changes in near real-time [5–10]. The reproductive number provides a interpretable indicator of epidemic dynamics: $R_e > 1$ corresponds to a growing outbreak while $R_e < 1$ corresponds to a declining outbreak. This threshold also gives an intuitive understanding of the reduction in transmission that is necessary for the epidemic to be curbed, which is particularly useful to public health authorities in epidemic contexts [11]. Moreover, $R_e$ estimates can be linked to changes in policy, population behavior and immunity, pathogen evolution, and other factors [1, 3, 12–14].

The COVID-19 pandemic revealed that pre-pandemic methods were not equipped to monitor ongoing outbreaks (as opposed to revisiting past outbreaks) or to deal with delayed and incomplete observations of infection events [1]. Thus, new methods were developed to fill this gap [15–18].

Here, we present estimateR, an R package to estimate $R_e$ from delayed and incomplete observations of infection events. This is the first package-based implementation of the methodology developed in Huisman et al. [18]. While their software pipeline was extensively used and tested during the COVID-19 pandemic, its implementation was not optimized for usability by third parties. Instead, the estimateR package offers a fully-documented and accessible implementation of the method to any R user. It was designed specifically for ease of use in a variety of infectious disease outbreak contexts. Because of its modularity, it can easily be extended as new $R_e$ estimation methods become available.

## Implementation

### Method summary

The estimateR R package provides tools to estimate the effective reproductive number in a timely fashion based on observational time series data from an epidemic. The core method implemented by estimateR is an improved version of the methodology developed for COVID-19 by Huisman et al. [18]. A full description of the method implemented in estimateR is provided in “Appendix A” section.

In brief, this method consists of 4 separate steps chained together to estimate $R_e$ and the associated 95% confidence interval from noisy and delayed observations of infection events. These observations include case confirmations, hospital admissions, intensive care unit admissions or deaths. The delay between an infection event and a recording depends on the observation type. In the first step, the input data is smoothed to reduce the effect of observation noise on the resulting $R_e$ estimates. “Noise” refers to any randomness that is not related to infection dynamics or stochastic reporting delays (e.g., from missing data, incomplete reporting, imported cases from abroad and other such sources of variability). Second, a time series of infection events is reconstructed from the smoothed observation data. Each observation is modelled as the result of an infection event combined with a waiting time drawn from a delay distribution (describing the time from infection to observation). To reconstruct the original series of infection events, the delay distribution is removed (deconvolved) from the observation data using an expectation-maximisation algorithm [19]. Third, $R_e$ is estimated from the inferred series of infection events, using the EpiEstim R package [8]. Finally, to estimate the uncertainty around the $R_e$ point estimates, bootstrap replicates are built from the original data. Each replicate goes through the three steps described above, allowing the construction of a confidence interval.

### Package structure

Each of the four analysis steps described above (1. smoothing, 2. deconvolution, 3. $R_e$ inference and 4. bootstrapping) is built as an independent module and can be used as a building block in an analysis pipeline. The standard use case, i.e. estimating $R_e$ from a time series of noisy and delayed observations of infection events, requires all four building blocks. However, we also accommodate different use cases: for instance, a user might be interested in recovering a time series of infection events rather than $R_e$ (i.e., using only steps 1, 2, 4) while another user may rely on incidence data that does not require smoothing (using only steps 2, 3, 4). The modular structure is complemented by a number of so-called “pipe functions”. Each of these functions corresponds to a particular type of analysis that can be carried out with estimateR.

Furthermore, within each module, one or multiple methods are provided for users to choose from. For instance the $R_e$ estimation module implements both an option to estimate $R_e$ as a continuous function of time and an option to estimate it as a piecewise constant function of time (step-function). In the future, we plan to continue to extend the possibilities offered by estimateR by implementing additional options for the various modules. Others are also invited to build on the existing code base by implementing new options, whether for their own use or for the community.

In summary, the code is structured to give as much freedom as possible to users and method developers, while providing sensible default configurations to ensure a high level of usability.

### Inputs and outputs

In the standard use case of estimateR, $R_e$ values are estimated from noisy delayed observations of infection events. Required inputs are a time series of observations, the generation time distribution of the outbreak (distribution of time elapsed between successive cases in a transmission chain), and the distribution of the delay between infection events and recorded observations. These delays can be expressed as a single probability distribution or can combine several independent delay distributions. For instance, the delay between infection and hospital admission may be broken down into two successive delays: one from infection to symptom onset (incubation period) and another from symptom onset to hospital admission.

The default output of an estimateR analysis is a dataframe containing $R_e$ estimates through time, along with 95% confidence interval boundaries. When relevant, a date of reference can be passed as input, corresponding to the date of the first recorded incidence. A date column is then included in the output. Optionally, results from intermediate steps of the analysis can also be included in the output.

There are many more inputs to the main estimateR functions. These are associated with sensible default values applicable to a wide range of use cases, and are well-documented to allow users to alter them when required. Specific use cases of estimateR may require adapted inputs. As estimateR can handle delay distributions that vary through time, the delay information can also be input as a table containing records through time of individually-recorded delays. Such a table can be derived from a line list of the outbreak of interest. This information can also be passed as a matrix specifying delay distributions through time. These options are described in more detail in the estimateR documentation.

### Handling issues relating to incomplete data

Epidemic case data is intrinsically complex, as the true infection time is often unknown and observed with a certain delay, and time series of observations may be truncated or incomplete. We describe three new features, implemented in estimateR to improve the method described by Huisman et al. [18] in the face of these issues.

#### Handling truncated incidence data

In some outbreaks, the window for which incidence data is available excludes the beginning of the outbreak. This may happen for a number of reasons. For instance, cases may not have been properly recorded and centralized before a particular date. Or public health authorities may change the way incidence is recorded at some point during an outbreak, rendering early data difficult to combine with newer data. To better handle such issues, whenever smoothing incidence data at the beginning of the time series, estimateR extrapolates incidence in the past assuming a growth rate corresponding to the observed average growth rate over the first few data points. This allows the smoothing function to reconstruct a trend at the beginning of the time series closer to the most plausible trend. To avoid biasing downstream computations, the extrapolated data points are discarded after the smoothing step (see “Appendix A” section for details).

#### Inference of the series of infection events

The deconvolution step to infer infection events from delayed observations is implemented using an expectation-maximisation algorithm. This algorithm iteratively improves on an initial guess for the time series of infection events. In estimateR this initial guess is built from the series of delayed observations shifted towards the past by a number of time steps. The gap left by this shift is filled by extrapolating the series of observations assuming a constant growth rate equal to the last observed rate (see “Appendix A” section for details).

#### Dealing with partially-delayed observations

In estimateR, when combining partially-delayed and fully-delayed observations (see “Appendix B” section for definition and details), the nowcasting of partially-delayed observations is performed before the partially-delayed series of observations is smoothed.

## Results and discussion

### Validation of the estimateR implementation on simulated data

#### Basic validation

**Fig. 1:**
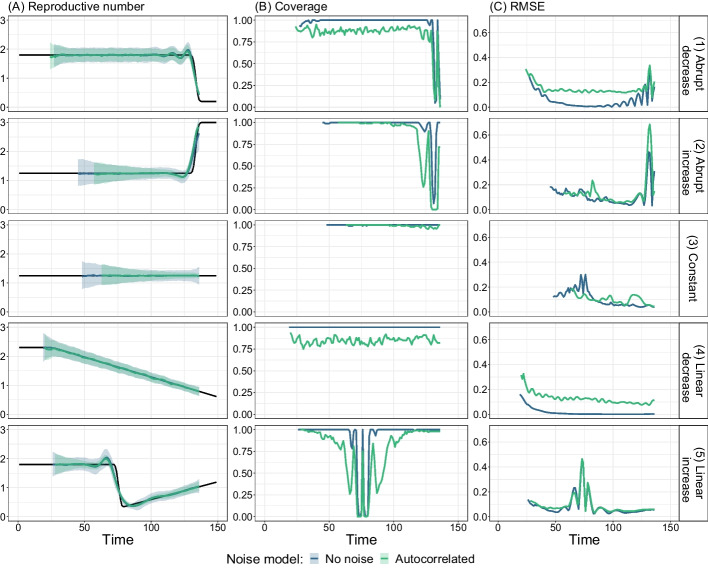
Summary of $R_e$ inference on simulated data. Each row corresponds to a different scenario of $R_e$ changes through time. Values shown in blue correspond to data simulated without additional observation noise whereas the green values correspond to data simulated with an auto-correlated noise model. The first column shows estimated $R_e$ values, with the ground truth as a black line. For each noise model, the median (over 100 replicates) estimate is shown as a line and the 95% confidence interval is shown as a ribbon. The second column shows corresponding coverage values (fraction of replicates for which the ground truth is inside the confidence intervals) and the third column shows the root mean squared error (RMSE)

To validate the implementation of estimateR we first tested its ability to monitor $R_e$ on a number of simulated scenarios. We simulated infection events through time, according to five representative trajectories the reproductive number could follow during an outbreak (see Fig. 1). These scenarios were designed to test how accurately the reproductive number is estimated (1) during phases when $R_e$ is constant or gradually changing, (2) when $R_e$ increases or decreases abruptly and (3) close to the present. The full simulation procedure is detailed in “Appendix C” section. For these simulations the delay from infection to observation was fixed through time and had a median of 14 time steps.

First, we considered a case without observation noise, with only Poisson noise from the infection process itself (see “Appendix C” section for details). This constitutes an ideal case where we expect $R_e$ estimation to work best and no smoothing step is necessary when estimating $R_e$. Results are summarized in Fig. 1, along with coverage of the 95% confidence intervals and the root mean squared error (RMSE). $R_e$ estimates are generally of good accuracy, with coverage close to 1, corresponding to a slight over-coverage. Abrupt changes in the true reproductive number are slightly smoothed over, which leads to a reduced coverage and higher RMSE in regions of abrupt changes. This slight smoothing is because $R_e(t)$ correspond to the average estimated $R_e$ over 3 time steps (see subsection *Estimation of the effective reproductive number* $R_e$ in “Appendix A” section).

We then considered a more realistic scenario by adding observation noise to the simulated observations. Auto-correlated noise was generated using an auto-regressive noise model of order 4 (AR(4)). The noise model and its coefficients were selected to approximate country-level empirical COVID-19 incidence data [18], where testing showed a clear weekly pattern. Now, observations were smoothed prior to the $R_e$ estimation. Again, the recovered $R_e$ estimates (column A) are highly similar to the true value assumed in the simulations. Compared to the scenario without noise, the coverage is slightly reduced and the error is slightly increased (e.g., a coverage of 0.85 rather than 1 in scenario with a linearly-decreasing $R_e$; Fig. 1 panel 4B). Overall, these simulations confirm the general validity of the estimateR implementation.

Note that, when using estimateR, it is not recommended to smooth observations that do not exhibit strong observation noise, as this decreases the ability to detect rapid changes in $R_e$ trends. Additional file 1: Fig. S1 shows the simulations without observation noise, where we estimated $R_e$ with and without an (unnecessary) smoothing step. We see that the unnecessary smoothing of observations causes a stronger smoothing of $R_e$ trends, resulting in comparably lower coverage of the estimates in time windows with abrupt $R_e$ changes (see Additional file 1: Fig. S1, scenarios 2 and 5). Similar conclusions were reached when testing the original software pipeline in Huisman et al. [18].

#### Validation on simulated data containing partially-delayed observations

We performed a variation on the simulation study presented above and investigate the effect of combining partially- and fully-delayed observations. As described in greater detail in “Appendix B” section, a pair of types of observations can be called “partially-delayed” and “fully-delayed” when one type of observations (the partially-delayed observations) is an intermediary step between infection and the other type of observations (the fully-delayed observations). For instance, onset of symptoms is often an intermediary step between infection and case confirmation. The advantage of partially-delayed observations is that they, by definition, are less delayed and thus allow for $R_e$ estimates closer to the present. In addition, the narrower delay distribution spreads out observations less and thus paints a less blurred picture of the underlying infection incidence.

We simulated pairs of partially-delayed and fully-delayed time series as described in “Appendix C” section. We tested four scenarios with varying fractions of partially-delayed observations *p*: 0, 0.3, 0.6, 1. The parameter setting $p=0$ corresponds to the scenario where we only had access to fully-delayed observations (e.g., only dates of case confirmations). Conversely, with $p=1$, we obtain a scenario where we have only partially-delayed observations (e.g., dates of symptom onset were recorded for all confirmed cases). Additional auto-correlated observation noise was included in this analysis.

**Fig. 2:**
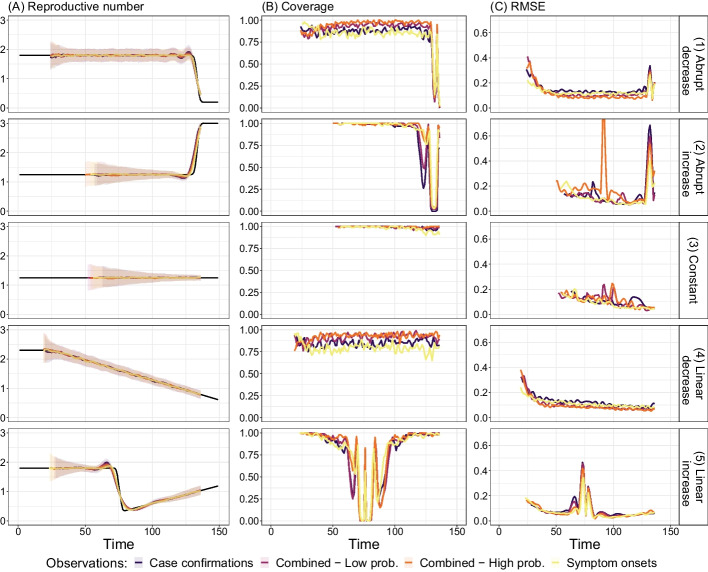
Summary of $R_e$ inference on simulated data combining partially-delayed and fully-delayed observations. Each row corresponds to a different scenario of $R_e$ changes through time. Each plot overlays values obtained on simulations obtained with four different values of *p* (probability of making a partially-delayed observation for a given infection event): from purple to yellow, $p = {0, 0.3, 0.6, 1}$. The first column shows estimated $R_e$ values, with the ground truth as a black line. For value of *p*, the median (over 100 replicates) estimate is shown as a line and the 95% confidence interval is shown as a ribbon. The second column shows the corresponding coverage values (fraction of replicates for which the ground truth is inside the confidence intervals) and the third column shows root mean squared error (RMSE)

From these simulated observations, we used estimateR to recover the dynamics of $R_e$ through time. Results (estimates, coverage and RMSE values) are summarized in Fig. 2. The higher the proportion of partially-delayed observations (e.g., symptom onsets) the better the $R_e$ estimates follow real $R_e$ values around abrupt $R_e$ changes, as seen in by the lower RMSE values (column C in Fig. 2; especially row 1, 2, and 5). The relative coverage is slightly lower for higher values of *p* in the first (stable period before $R_e$ drop) and fourth scenario, but RMSE values do not increase compared to lower values of *p*. The decreased coverage seems to be attributable to slightly more jittery $R_e$ estimates as *p* increases, which could be addressed by increasing the smoothing parameter $\sigma$ (see “Appendix A” section for additional details). Overall, when partially-delayed observations are available, including them can improve the $R_e$ estimation during periods of rapid $R_e$ changes. Precision in estimates during these periods is particularly relevant to outbreak monitoring.

#### Validation on simulated data generated with time-varying delay distributions

Finally, we investigated the effect of time-varying delay distributions on the estimation of $R_e$. Delays between infection events and case observations can shorten or lengthen throughout the course of an outbreak [18], and estimateR can account for these variations.

To test and validate this capability, we simulated outbreaks with time-varying delay distributions, as described in “Appendix C” section. The delay from infection to observation gradually changed from a short to a long delay (or vice versa) over the course of the simulated outbreak. Auto-correlated observation noise was added to the simulated observations. We then estimated $R_{e}$ from the simulated case incidence, using either a constant delay distribution (corresponding to the delay distribution at the start of the outbreak or at present time) or the correct time-varying distribution.

**Fig. 3:**
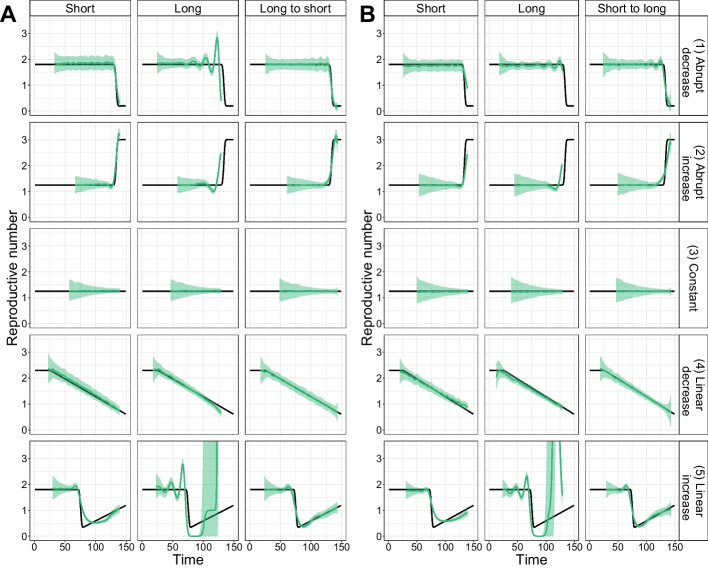
Summary of $R_e$ inference on simulated data with time-varying delay distributions. **A** $R_e$ estimates on simulated data, with observation delays gradually changing from a long (at time 0) to a short (at time 150) observation delay distribution. **B** $R_e$ estimates on simulated data, with observation delays gradually changing from a short (at time 0) to a long (at time 150) observation delay distribution. **A** and **B** Each row corresponds to one of five $R_e$ scenarios. Each column corresponds to a different delay distribution in the analysis. In the first two columns, delay distributions are fixed and either short or long. In the third column, delay distributions are allowed to vary when estimating(from short to long or long to short). In each plot, the ground truth $R_e$ is shown as a black line, the median (over 100 replicates) estimate is shown as a green line and the 95% confidence interval is shown as a green ribbon

We summarise the $R_e$ estimates in Fig. 3 and report coverage and RMSE values in Additional file 2: Fig. S2. When estimating $R_{e}$ with correctly specified, time-varying delay distributions (last column in Fig. 3, panels A, B), $R_e$ estimates behave well for the entire time span. Instead, when $R_{e}$ is estimated with a constant delay distribution, this constitutes a method misspecification with respect to the simulations. As a result, the estimates are only accurate for the time period where the constant delay distribution is close to the time-varying one (e.g., the short delay distribution is similar to the start of a simulation with a delay distribution varying from short to long, Fig. 3 panel B). To our knowledge, estimateR is the only package that allows the specification of time-varying delay distributions in the estimation, and thus to avoid such bias.

In summary, our simulation study demonstrates the validity of the estimateR implementation. Results obtained are in line with those presented on the original implementation of the Huisman et al. [18] method. Estimates are accurate, both in reconstructing past outbreak dynamics and close to the present, which highlights the suitability of estimateR for outbreak monitoring. Nevertheless, simulations also show the limitations previously described for this method [18]: we observed situations of over- and under-coverage and the smoothing required to account for the observation noise can smooth abrupt variations in $R_e$.

### Improvements to the method of Huisman et al.

In the “Implementation” section, we described three improvements estimateR made over the Huisman et al. implementation for handling incomplete data. For each of these features, we compared $R_e$ estimates obtained with the estimateR method and the Huisman et al. method. Simulations were performed as above (see “Appendix C” section for details) with the same parameter values for both methods and including auto-correlated observation noise.

#### Handling truncated incidence data

**Fig. 4:**
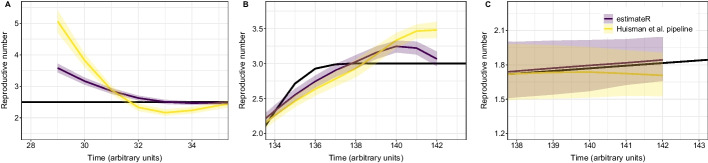
Impact of method improvements. Each panel shows the impact of one of the three method alterations, by summarizing $R_e$ estimates over 100 simulated replicates. In each plot, the ground truth $R_e$ is shown as a black line, and the median estimate is shown in dark purple and yellow respectively for estimateR and the Huisman et al. method. The coloured ribbons are bounded by median confidence interval boundaries over 100 replicates. **A** Early estimates for truncated incidence data. **B** Most recent estimates when using or not using the latest trend in the deconvolution step. **C** Most recent estimates nowcasting before or after smoothing partially-delayed observations

To investigate the impact of extrapolating observation counts that were truncated off, we assumed a constant $R_e$, simulated 100 outbreaks and truncated the simulated observations, removing all data points before the 30th time step (Fig. 4A). Early values of $R_e$ are difficult to estimate because an important part of the data informing these estimates is missing. The results show that early $R_e$ values are overestimated compared to the ground truth. Still, these estimates are less biased with estimateR than with the Huisman et al. method.

#### Inference of the series of infection events

To investigate the impact of extrapolating future observations in the initial step of the deconvolution algorithm, we assumed a sharply increasing $R_e$ before a stabilization close to the present—similar to the “Abrupt increase” scenario of our simulation study—and focused on the most recent $R_e$ estimates from both implementations (Fig. 4B). $R_e$ estimates are close to the ground truth with estimateR whereas a stronger upward bias is observed with the Huisman et al. method.

#### Dealing with partially-delayed observations

Finally, we investigated the impact of nowcasting unseen partially-delayed observations before smoothing instead of after. To do so, we performed simulations of partially-delayed and fully-delayed observations with $p=1$: all infections have an associated partially-delayed observation. We assumed a reproductive number evolving as in the “Linear increase” scenario of our simulation study, and report results in Fig. 4C. We observe a downward bias on $R_e$ estimates with the Huisman et al. method, whereas no such bias appears with estimateR.

### Comparison with other methods

#### Comparison on simulated data

We compared the accuracy of estimates from estimateR against epidemia [15] and EpiNow2 [16], two prominent and recently-developed R packages for $R_e$ estimation, on our simulated data.

As before, we simulated outbreaks following five different $R_e$ trajectories to compare performance in different contexts (simulation details in “Appendix C” section). We restricted the analysis to 50 replicates (instead of 100) for epidemia and EpiNow2 due to the time taken by computations. We could not generate meaningful results with either package on simulated data with an auto-correlated observation noise model, as used in the simulation study above. Thus, we used log-normal distributed multiplicative noise instead, with independent values drawn from one time step to the next. Parameter specifications are listed in Table 1.

**Table 1:** Parameter values used for method comparison

R package	Parameter	Value	Notes
estimateR	Smoothing parameter $\sigma$	9 Time steps	
	Incubation period	Gamma (shape=3.2, scale = 2.1)	As specified in simulations
	Observation delay	Gamma (shape=2.7, scale=2.6)	As specified in simulations
	(from symptoms to confirmation)		
	Generation time	Gamma (mean = 4.8, SD = 2.3)	As specified in simulations
	Other parameters	Default settings	
epidemia	Generation time	Gamma (mean = 4.8, SD = 2.3)	As specified in simulations
	Observation model family	Negative binomial	Analyses failed with log-normal
			(log-normal fits
			simulated noise)
	Delay distribution	Discretized convolution of	As specified in simulations
	(from infections to observations)	Gamma (shape=3.2, scale = 2.1)	
		and	
		Gamma (shape=2.7, scale=2.6)	
	Hyperprior scale	0.2	
	on $R_t$ random walk		
	Other parameters	Default settings	
EpiNow2	Incubation period	Log-normal($\mu$ = 1.68, $\sigma$ = 0.63)	Log-normal fit of
			Gamma (shape=3.2, scale = 2.1)
			(used in simulations)
	Observation delay		
	(from symptoms to confirmation)	Log-normal($\mu$ = 1.68, $\sigma$ = 0.67)	Log-normal fit of
			Gamma (shape=2.7, scale = 2.6)
			(used in simulations)
	Generation time	Gamma(mean = 4.8, SD = 2.3)	As specified in simulations
	Gaussian process	Applied to global mean	
	Observation model family	Negative binomial	
	Other parameters	Default settings	

**Fig. 5:**
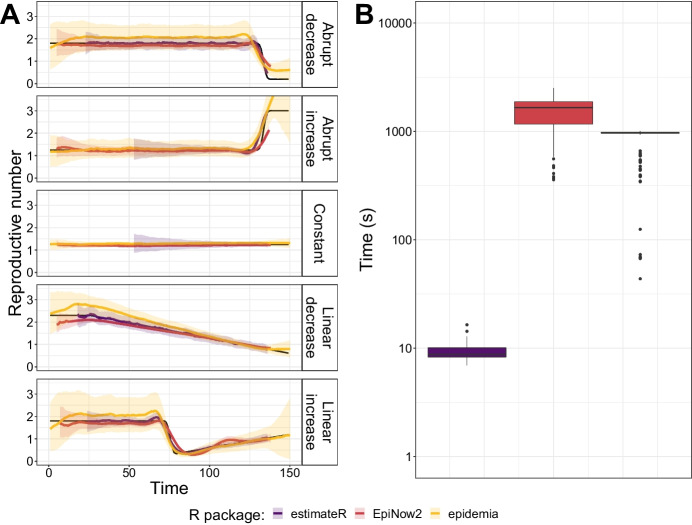
Comparison of $R_e$ inference on simulated data for three software packages: estimateR, epidemia and EpiNow2. **A** $R_e$ inference results. Each row corresponds to a different scenario of $R_e$ changes through time. The ground truth is shown in black, estimateR, epidemia and EpiNow2 estimates are in blue, green and red, respectively. For each method, the median of point estimates is shown as a line and the ribbon is bound by the median of the lower and upper confidence interval boundaries over the analysed replicates (100 replicates for estimateR, 50 for epidemia and EpiNow2). **B** Computation time (on a log scale) required to complete the $R_e$ estimation process on one simulated data replicate

The results of our comparison are summarized in Fig. 5. Figure 5A presents the median of mean estimates and 95% confidence intervals across all analyzed replicates. For EpiNow2, we only show non-nowcast results for easier comparison with estimateR. Performance metrics (coverage and RMSE) are plotted in Additional file 3: Fig.  S3.

On this simulated data, all three methods yield comparable results, with estimateR performing most accurately. It achieves a consistently high coverage and low error, and more accurately follows abrupt $R_e$ changes than the other two packages, both in the past and close to the simulated present-time. We find that epidemia overestimates $R_e$ in parts of the first, fourth and fifth scenarios whereas EpiNow2 slightly underestimates $R_e$ in parts of the first, fourth and fifth scenarios. Moreover, epidemia uncertainty intervals are very wide, leading to an over-coverage (coverage above 0.95 for a 95% confidence interval; Additional file 3: Fig. S3A) for some data windows.

We note that a certain degree of model misspecification could explain the comparatively worse performance of epidemia and, to a lesser extent, of EpiNow2. For both packages, we specified negative binomial observational models, whereas noise in the simulated data results from Poisson noise when generating infections combined with log-normal noise when generating observations. epidemia offers the option to specify a log-normal observation model, but we did not manage to set up an analysis with this option (the inference either failed or returned diverging $R_e$ values). This model misspecification is likely the cause of performance issues observed. We note that estimateR assumes auto-correlated observation noise, and thus the estimateR analysis is also misspecified.

#### Speed comparison

In addition to comparing estimated values, we compared the computation speed of the three methods. Figure 5B shows the distribution of computing time observed when estimating the reproductive number on a single simulated time series of observations. The observations were made during the computation of estimates presented in panel A. Here we find estimateR to be considerably faster than both epidemia and EpiNow2. In our simulation study (on a MacBook Pro, with a 2.3 GHz Dual-Core Intel Core i5, with 4 logical CPU cores), analyzing a time series of observations took on average 9 s with estimateR, whereas it took 14 min (850 s) with epidemia and 25 min with EpiNow2 (1520 s).

The need for epidemia and EpiNow2 to carry out Bayesian posterior distribution sampling via Markov chain Monte Carlo likely explains why estimateR is much faster in comparison. Indeed, the computations performed by estimateR are much simpler and much less computationally-intensive, as they do not involve any sampling of posterior distributions. In particular, estimateR makes use of EpiEstim for the final *Re* estimation step, taking advantage of the analytic solution derived for the posterior distribution of $R_e$ by Cori et al. [8].

We note that this comparison only provides a qualitative evaluation of differences in speed, as the computational effort to run each method can vary with the specific data and estimation settings. For example, we here used the default approach of estimating uncertain delay distributions in EpiNow2, while it is also possible to fix the delay distributions to speed up computation. On the other hand, it should be noted that we ran the Markov chains in epidemia and EpiNow2 with 4 cores in parallel, while estimateR only requires a single core. Thus, when using estimateR, one could use e.g. 4 cores at once to estimate the reproductive number of 4 different time series in parallel, further increasing the speed advantage of the package.

#### Feature comparison

Like epidemia [15] and EpiNow2 [16], estimateR accounts for delays between infection events and observations, which is essential for outbreak monitoring [1]. In contrast however, estimateR also allows for delay distributions that vary through time, and can directly combine incidence data from partially-delayed and fully-delayed observations. As demonstrated in simulations, both of these features improve the accuracy of the estimates. In general, the availability of high-quality data, in particular of line lists rather than aggregated data, is necessary to harness the power of these features. While EpiNow2 can directly integrate uncertainty of user-specified delay distributions in its model [16], such uncertainty must rather be accounted for through sensitivity analyses when using estimateR. Moreover, in contrast to the epidemia and EpiNow2 packages, estimateR does not permit any forecasting of future epidemic dynamics [15, 16].

### Application to empirical data

#### COVID-19

**Fig. 6:**
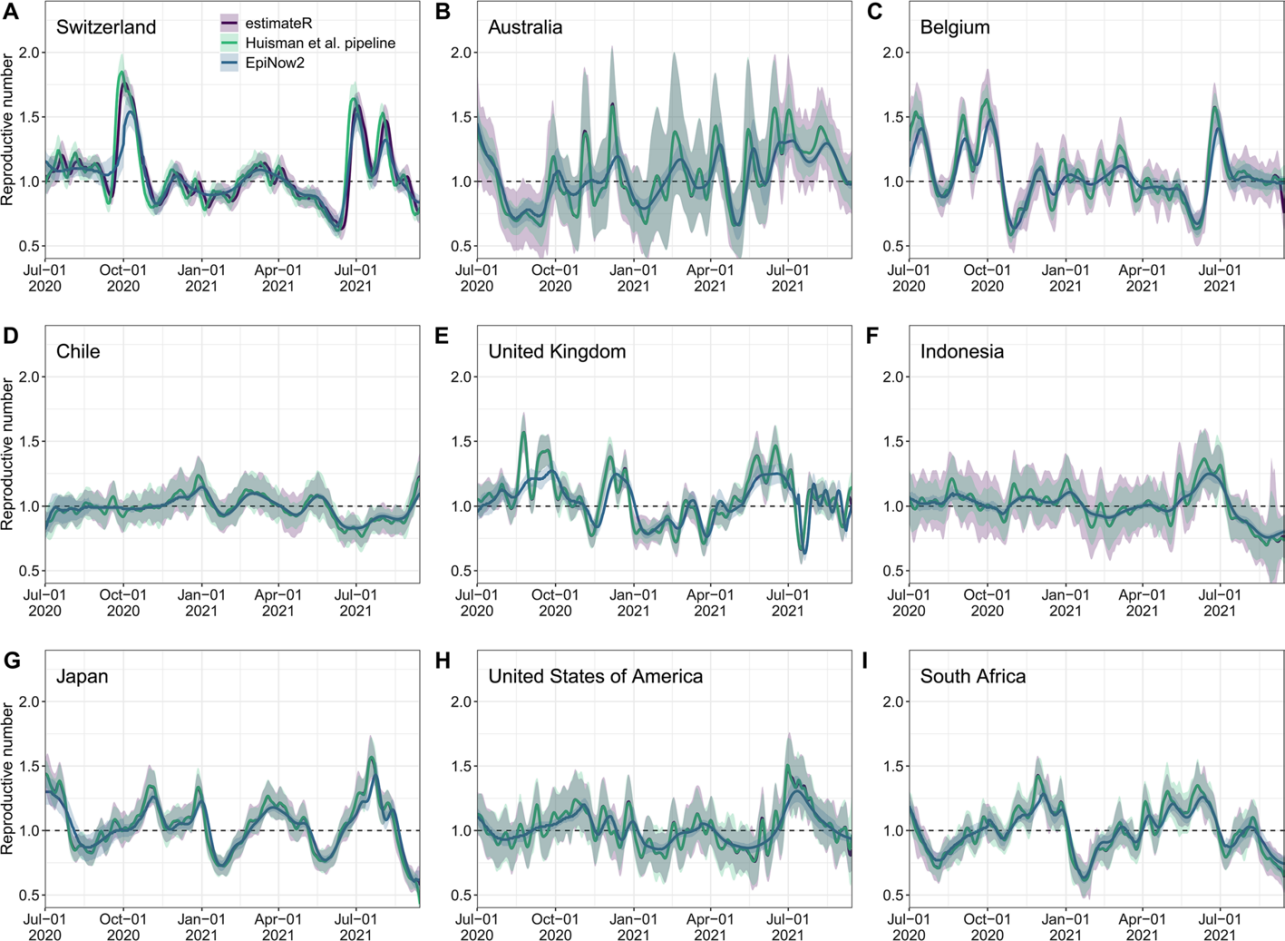
$R_e$ estimates through time on COVID-19 case data (between July 1, 2020 and September 15, 2021) from nine countries (**A**–**I**). Each plot shows point estimates (lines) and uncertainty intervals (ribbons) from estimateR (purple), the Huisman et al. software pipeline (green), and EpiNow2 (blue)

To test estimateR on empirical data, we analysed COVID-19 incidence data from 9 countries between July 1, 2020 and September 15, 2021 using estimateR. We compared the results with publicly available estimates by Huisman et al. [18], which were produced during the COVID-19 pandemic (Fig. 6). The analyses for estimateR were parameterized with the same serial interval and delay distributions as described in Huisman et al. As expected, estimateR produced estimates very similar to the pipeline by Huisman et al., which has the same underlying methodology. Minor differences observed are due to the method improvements described above. In particular, differences are most pronounced for Switzerland (Fig. 6A), where line list data were available to estimate $R_e$. The different ways of extracting the time-varying delay distributions from the line list led to slight discrepancies between estimateR and Huisman et al. (details for estimateR are described in “Appendix A” section). For all other countries, no line list data was available and constant delay distributions were assumed.

For comparison, we also obtained estimates from the publicly available EpiForecasts dashboard by Abbott et al. [20], which uses EpiNow2 as an underlying $R_e$ estimation method [16]. The trend of the EpiForecast estimates qualitatively agrees with the estimates from estimateR and Huisman et al., however they are generally less volatile and have lower uncertainty. Such differences likely result from the different approaches to smoothing case counts and $R_e$ estimates, as well as the default values used for method hyperparameters. There is currently no way to know how smoothly $R_e$ varies during real infectious disease outbreaks. By running these two $R_e$ estimation packages side by side, researchers can study multiple hypotheses and ultimately reach a deeper understanding of the underlying disease transmission dynamics.

#### Dengue fever

**Fig. 7:**
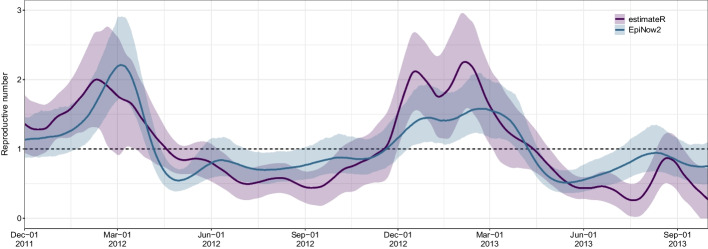
$R_e$ estimates through time on dengue fever case data (between December 1, 2011 and October 1, 2013) from Rio de Janeiro, Brazil. Shown are point estimates (lines) and uncertainty intervals (ribbons) obtained with estimateR (purple) and EpiNow2 (blue)

As an example of an endemic disease with seasonal patterns and indirect transmission mechanism, we further applied estimateR to incidence data from two seasonal waves of dengue fever in Rio de Janeiro, Brazil (between December 1, 2011 and October 1, 2013). Here we used incubation period and generation interval distributions from the literature [21, 22], and an empirical reporting delay distribution as estimated from line list data [23] (see “Appendix E” section for details). For comparison, we also produced estimates using EpiNow2, with the same delay distribution and epidemiological parameters as for estimateR. As shown in Fig. 7, both methods clearly track the two seasonal waves observed in the analyzed time frame, with $R_e$ estimates significantly above the exponential threshold of 1. During the 2011/2012 seasonal wave, estimates from both approaches generally agreed in the magnitude and trend of $R_e$, with estimateR inferring a slightly earlier and more uncertain peak in $R_e$ than EpiNow2. In the 2012/2013 wave, estimateR inferred considerably higher $R_e$ values than EpiNow2, however both approaches agreed closely on the start and end date of the exponential growth phase (timing of $R_e$ crossing the threshold of 1). In between the two seasonal waves, estimateR produced estimates more confidently below the epidemic threshold EpiNow2.

#### Influenza in wastewater

In addition to incidence data collected by public health authorities, estimateR can also be used to estimate the effective reproductive number from longitudinal measurements of virus in wastewater. After establishing this use case for SARS-CoV-2 [24], we have extended the work to monitor the dynamics of seasonal influenza in the wastewater of three major Swiss cities and compared it to estimates obtained from influenza case data [25].

### Limitations

The estimation method implemented in estimateR is subject to known limitations [1, 18]. In particular, we emphasize that properly accounting for the specific transmissibility of imported cases can be important when a large fraction of cases recorded are not local cases [26]. Like EpiEstim, estimateR can account for a segregation of local and imported cases whereby imported cases do not result from infection by existing local cases, but contribute to future infections. Unlike the method presented by Tsang et al. [26], estimateR does not allow for a difference in transmissibility between local and imported cases.

In its current version, estimateR can only handle non-negative delay distributions which can be a limitation when handling specific types of observed events (such as pre-symptomatic case observations). Moreover, estimateR makes strong simplifying assumptions on the outbreak studied. First, it assumes a constant serial interval when estimating $R_e$ from reconstructed infection events [8], whereas relaxing this assumption can improve estimates [1, 10]. Also, a constant ascertainment rate is assumed for all observations. When the ascertainment rate changes in time, $R_e$ estimates are unreliable until the ascertainment rate is stable again.

## Conclusions

We present estimateR, an R package for estimating the reproductive number through time from incidence data. This software is a new and improved implementation of the $R_e$ estimation pipeline in Huisman et al. [18]. Compared with two existing software packages, estimateR is substantially faster and more accurate in the tested simulation scenarios. estimateR offers simple-to-use functions to monitor an ongoing outbreak, to revisit past outbreaks, and to inform epidemic models that require $R_e$ estimates as input. With its modular design, it exposes the inner steps of the analysis; more experienced users can use these functions as building blocks, combining them or using them individually in their own analyses. The package is structured to make it as simple as possible for users to implement their own extensions and upgrades. Our goal is that estimateR can serve as a collaborative tool for the scientific community.

### Supplementary Information

**Additional file 1: Fig. S1**. Summary of Re inference on simulated data without added observation noise, obtained with and without an initial smoothing step. Each row corresponds to a different scenario of Re changes through time. The first column shows the ground truth as a black line, and the median (lines) and lower and upper bounds of the 95% confidence interval (ribbons) of Re estimates obtained over 100 replicates, with (purple) and without (blue) an initial smoothing step, respectively. The second column shows corresponding coverage values (fraction of replicates for which the ground truth is inside the confidence intervals) and the third column shows root mean squared error (RMSE) values for each scenario.

**Additional file 2: Fig. S2**. Coverage and RMSE values on Re estimates on simulated data with time-varying delay distributions. Each row corresponds to one of five Re scenarios. Each column corresponds to a different delay distribution in the analysis. In the first two columns, delay distributions are fixed and either short or long. In the third column, delay distributions are allowed to vary when estimating(from short to long or long to short). A and C: Coverage and RMSE values on Re estimates on simulated data with observation delays gradually changing from a long (at time 0) to a short (at time 150) observation delay distribution. B and D: Coverage and RMSE values on Re estimates on simulated data, with observation delays gradually changing from a short (at time 0) to a long (at time 150) observation delay distribution.

**Additional file 3: Fig. S3**. Coverage and Root Mean Squared Error of Re estimates using estimateR, epidemia and EpiNow2. The rows show five scenarios of Re variations through time. A: Coverage values (fraction of replicates for which the ground truth is inside the confidence intervals). B: Root Mean Squared Error (RMSE) values.

## Data Availability

The estimateR code source, with instructions for the package installation, is available at https://github.com/covid-19-Re/estimateR. The package documentation, including vignettes to get started, is available at https://covid-19-re.github.io/estimateR/. Data and scripts to reproduce all analyses and figures presented in the manuscript are available at https://github.com/jscire/estimateR_paper_code. The accompanying zip file contains a snapshot of the estimateR software repository (https://github.com/covid-19-Re/estimateR) and a snapshot of the online repository containing all data files and scripts used to produce the analyses presented in this manuscript (https://github.com/jscire/estimateR_paper_code/). Both snapshots date from May 29, 2023. • **Project name:** estimateR. • **Project home page:** (https://github.com/covid-19-Re/estimateR). • **Operating systems:** Platform independent. • **Programming language:** R. • **Other requirements:** R 2.1 or higher. • **License:** GNU GPL 3. • **Any restrictions to use by non-academics:** as specified by license.

## Appendix

### Appendix A: method description

This section contains a full description of the base method implemented in estimateR. This method was developed by Huisman et al. and the text of Appendix A is adapted from the original method publication [18], with modifications specific to estimateR (main modifications are listed in the “*Handling issues relating to incomplete data*” section of the main text).

#### Smoothing of noisy observations

To smooth the incidence data, estimateR implements local polynomial regression (LOESS). By default, estimateR performs LOESS smoothing with 1st order polynomials and a smoothing parameter $\sigma$ set such that 21 time steps in the local neighbourhood of each point are included.

Importantly, $\sigma$ should be adapted by estimateR users to the level of noise observed in their raw incidence data. This can be done by smoothing the raw observations with varying $\sigma$ values until the smoothed trend matches expectations.

Before smoothing, the raw time series of observations $(O_0, \dots , O_N)$ is padded at its left boundary with values extrapolating the initially observed trend (see the “*Handling issues relating to incomplete data*” section of the main text). To extrapolate these values, we first compute the average ratio between the incidence observed on a time step and the previous time step:

1 $$\begin{aligned} a&= \frac{1}{n} \sum _{i = 0}^{n-1} \frac{O_{i+1}}{O_i}, \end{aligned}$$

*n* being the number of time steps included in this average, by default it is set to 5 in estimateR.

Then, we build the padding values $(O_{-y}, \dots , O_{-1})$ by

2 $$\begin{aligned} O_{-i}&= O_0 \times a^{-i}. \end{aligned}$$

The number of padding values *y* is proportional to the length of the raw time series *N* and to the smoothing parameter $\sigma$.

After padding, LOESS smoothing is applied, and the smoothed values $(S_{-y}, \dots , S_{-1})$ are discarded to keep $(S_{0}, \dots , S_{N})$, the smoothed observations. Finally, the smoothed observations are normalised so that their sum is equal to the total number of raw observations ($\sum _{i\ge 0} O_i$).

#### Estimation of the infection incidence through deconvolution

To recover the non-observed time series of infection incidence from a time series of (optionally-smoothed) observations, estimateR implements a deconvolution algorithm. This algorithm deconvolves the time series of observations with a delay distribution specific to the type of observations (case confirmations, hospital admissions, deaths), to recover an estimate of the time series of infection events. It is an expectation-maximisation algorithm, generalised from the description made by Goldstein et al. [19], which is itself an adaptation of the Richardson-Lucy algorithm [27, 28].

Formally, the method infers a deconvolved output time series $(\lambda _{1}, \dots , \lambda _{N})$ from an input time series $({\bar{D}}_{K}, \dots , {\bar{D}}_{N})$, where $K \ge \mu$ ($\mu$ being the median of the delay distribution) and ${\bar{D}}_{i}$ indicates the (smoothed) number of observations on time step *i* (e.g., confirmed cases, hospitalisations, or deaths). Let $m^{j}_{l}$ be the probability that an infection on time step *j* takes $l\ge 0$ time steps to be observed. If no time-variation of the delay distribution is assumed $m^{j}_{l} = m_{l}$. Let $q_{j}$ be the probability that an infection that occurred on time step *j* is observed during the time-window of observations, i.e. is counted towards $({\bar{D}}_{K}, \dots , {\bar{D}}_{N})$. Then:

3 $$\begin{aligned} q_{j}&= \sum _{l = K - j}^{N - j} m^{j}_{l}\,. \end{aligned}$$

Let $E_i$ be the expected number of observed cases on time step *i*, for a given infection incidence $(\lambda _k)$:

4 $$\begin{aligned} E_{i}&= {\left\{ \begin{array}{ll} \sum _{j = 1}^{i} \lambda _{j} \, m_{i-j}^j &{} \text {for} \quad K \ge i \ge N\\ 0 &{} \text {for} \quad 0< i < K\,. \end{array}\right. } \end{aligned}$$

The deconvolution algorithm uses expectation maximisation [29] to find a final infection incidence estimate, which has the highest likelihood of explaining the observed input time series. To do so, it starts from an initial guess of the infection incidence time series $\Lambda ^{0} = (\lambda _{1}^{0}, \dots , \lambda _{N}^{0})$, used to compute $E_{i}^{0}$ according to Eq. 4, and updates the estimate in each iteration *n* according to the following formula:

5 $$\begin{aligned} \lambda ^{n+1}_{j}&= \frac{\lambda ^{n}_{j}}{q_{j}} \cdot \sum _{i = K}^{N} \frac{m_{i-j}^j {\bar{D}}_{i} }{E^{n}_{i}}\,. \end{aligned}$$

The iteration proceeds until a termination criterion is reached. Here, we follow Goldstein et al. and iterate until the $\chi ^{2}$ statistic drops below 1 [19]:

6 $$\begin{aligned} \chi ^{2}&= \frac{1}{N - K + 1} \sum _{i = K}^{N} \frac{(E^{n}_{i}-{\bar{D}}_{i})^{2}}{E^{n}_{i}}\,, \end{aligned}$$

or 100 iterations have been reached.

For the initial estimate of the incidence time series $\Lambda ^{0}$, the time series of observations is shifted backwards in time by the median of the delay distribution $\mu$. However, this leaves a gap of unspecified values at the start and end of the time series $\Lambda _0$. We augment the shifted time series with the first observed value (${\bar{D}}_K$) on the left. On the right side, we replace the missing values with an extrapolation of future observations. This extrapolation is specific to estimateR; it is done as follows:

7 $$\begin{aligned} \lambda _{N - i}&= {\bar{D}}_N \times \left( \frac{{\bar{D}}_N}{{\bar{D}}_{N - 1}}\right) ^{\mu - i}, \end{aligned}$$

for $$0 \le i < \mu$$.

*Time-varying delay distributions* When information on the time variation of delays between symptom onset and observation is available (e.g., through a line list), estimateR can take it into account during the deconvolution step. In this explanation, we need to break down the delay from infection to observation into two successive delays: an incubation period, which we assume to be fixed in time for simplicity, and a delay from onset of symptoms to observation which we allow to vary through time.

Recall that $m_{\ell }^j$ is the probability that an event occurring at time *j* (corresponding here to the onset of symptoms at time *j*) takes $\ell$ time steps to be observed. The $(m_{0}^j, \dots , m_{\ell _{max}}^j)$ time-varying delay distributions from onset of symptoms to observation are determined as follows: for each date *j*, the $n_0$ most recent recorded delays between symptom onset and observation, with onset date before *j*, are taken into account; $\ell _{max}$ being the highest observed delay (over all time steps). In estimateR, $n_0$ is, by default, at least 500 and up to 20% of all observations (both are flexible parameters).

The incidence data is right-truncated, meaning that, close to the present, hosts with recent onset of symptoms and with longer delay until observation have not been captured yet. Thus, the raw distribution of observed delays is biased towards shorter delays close to the present. To circumvent this effect, we fix the distribution for the reporting delay ($m_{\ell }^j$) after a certain time step *j*, so that delay distributions are not downward biased for infection dates close to the present. Let $({\bar{m}}_{0}, \dots , {\bar{m}}_{\ell _{max}})$ be the overall empirical delay distribution (aggregated over the entire window of observations) and *n* the 99$^{th}$ percentile of this distribution (*n* is the smallest integer for which $\sum _{i = 1}^{n}{\bar{m}}_i \ge 0.99$). For symptom onset dates *z* that are closer to the present than *n* (i.e., $N - z < n$, where *N* is the index of the last available data point), we fix $(m_{0}^{z}, \dots , m_{\ell _{max}}^{z})$ to be equal to $(m_{0}^{N - n}, \dots , m_{\ell _{max}}^{N - n})$.

Finally, the fixed incubation period and the time-varying delay from symptom onset to observation are convolved to generate a time-varying delay distribution from infection to observation.

#### Estimation of the effective reproductive number $R_{e}$

estimateR implements a wrapper around the method developed by Cori et al. [8], implemented in the EpiEstim R package, to estimate $R_{e}$ from a time series of infection events.

Disease transmission is modelled with a Poisson process. An individual infected at time $t-s$ is assumed to cause new infections at time *t* at a rate $R_{e}(t) \cdot w_{s}$, where $w_{s}$ is the value of the infectivity profile *s* time steps after infection. The infectivity profile sums to 1, and can be approximated by the (discretised) serial interval distribution [8]. The likelihood of the incidence $I_{t}$ at time *t* is thus given by:

8 $$\begin{aligned} P(I_{t} | I_{0}, \dots , I_{t-1}, R_{e}(t))&= \frac{\left( R_{e}(t)\Lambda _{t}\right) ^{I_{t}} e^{-R_{e}(t)\Lambda _{t}}}{I_{t}!}\,, \end{aligned}$$

9 $$\begin{aligned} \text {where}\hspace{1cm}\Lambda _{t}&= \sum ^{t}_{s=1} I_{t-s}w_{s}. \end{aligned}$$

The $R_{e}$ inference is performed in a Bayesian framework, and an analytical solution can be derived for the posterior distribution of $R_{e}(t)$ (see [8]; Web Appendix 1). By default in estimateR, the prior on $R_{e}(t)$ is a gamma distribution with mean 1 and standard deviation 5. The mean of the posterior distribution of $R_{e}$ is reported as being the point estimate.

Two options are available to estimate $R_{e}$: either it is treated as gradually changing through time or it is treated as a step-wise function of time. In the former case, the reported $R_{e}$ estimate for time step *T* summarises the average estimated $R_{e}$ over a period of $\tau$ time steps ending on time step *T*. By default in estimateR, $\tau = 3$. In the latter, $R_{e}$ is assumed to be constant on a number of intervals spanning the entire epidemic time window. The boundaries of these intervals must be given as user input.

#### Uncertainty estimation

To account for the uncertainty in the raw case observations, a 95% bootstrap confidence intervals is constructed for $R_{e}$. First, the case observations are re-sampled as follows: given the original case observations $D_t, t=K,\dots , N$, LOESS smoothing is applied to the log-transformed data $log(D_t+1)$ to obtain the smoothed values ${\hat{h}}_t$ and additive residuals $e_t$. Here log-transformation is used to stabilise the variance of the residuals.

From $e_t$, residuals are re-sampled to get $e^*_t$. This is done by an overlapping block bootstrap method to account for the time series nature of the data. Specifically, given the original residuals $(e_K, \dots , e_N)$, we first sample a block $(e^{*1}_1, \dots , e^{*1}_b)$ with default block length $b=10$. Weekly patterns in case observations can optionally be accounted for, if relevant. If so, the sampled block is built to start on the same day of the week (e.g., Tuesday) as the original case observations $D_K$. That is, we keep the longest part $(e^{*1}_{m_1}, \dots , e^{*1}_b)$ from $(e^{*1}_1, \dots , e^{*1}_b)$ such that $e^{*1}_{m_1}$ has the same day of the week as $D_K$. Then, we sample a new block $(e^{*2}_1, \dots , e^{*2}_b)$ and keep the longest part $(e^{*2}_{m_2}, \dots , e^{*2}_b)$ of $(e^{*2}_1, \dots , e^{*2}_b)$ such that the corresponding day of $e^{*2}_{m_2}$ follows on $e^{*1}_b$ (i.e. has the next day of the week if weekly patterns are accounted for). We glue these two sampled blocks together to get the temporal re-sampled residuals $(e^{*1}_{m_1}, \dots , e^{*1}_b, e^{*2}_{m_2}, \dots , e^{*2}_b)$. We repeat this process of adding blocks until the length of the re-sampled residuals is equal to or larger than the original residuals. In the latter case, we cut the last part of the re-sampled residuals to make sure its length is the same as the original residuals.

Finally, the bootstrap case observations are obtained by

10 $$\begin{aligned} D_t^* = \max (\exp ({\hat{h}}_t + e^*_t) -1, 0). \end{aligned}$$

The smoothing-deconvolution-estimation method is applied to the bootstrap case observation to obtain an estimate for $R_{e}(t)$, denoted by ${{\hat{\theta }}}^*(t)$. By repeating the above steps *B* times ($B = 100$ by default), we obtain ${{\hat{\theta }}}^*_{1}(t), \dots , {{\hat{\theta }}}^*_{B}(t)$. Then, we construct a Normal based bootstrap confidence interval for each time point *t* by:

11 $$\begin{aligned} \left[ {{\hat{\theta }}}(t) - q_z \left( 1-\frac{\alpha }{2}\right) {\widehat{sd}}({{\hat{\theta }}}^*(t)), \ {{\hat{\theta }}}(t) + q_z \left( 1-\frac{\alpha }{2}\right) {\widehat{sd}}({{\hat{\theta }}}^*(t)) \right] , \end{aligned}$$

where ${{\hat{\theta }}}(t)$ denotes the estimated $R_{e}(t)$ based on the original case observations, $q_z(1-\frac{\alpha }{2})$ denotes the $1-\frac{\alpha }{2}$ quantile of the standard normal distribution, and ${\widehat{sd}}({{\hat{\theta }}}^*)$ denote the empirical standard deviation of ${{\hat{\theta }}}^*_{1}(t), \dots , {{\hat{\theta }}}^*_{B}(t)$, (by default $\alpha =0.05$, to obtain 95% confidence intervals).

An implicit assumption for the above bootstrap confidence interval to be reasonable, is that the variance of the residuals $e_t$ is a constant over time *t* and does not depend on the value of the log-transformed data $log(D_t+1)$. This assumption roughly holds when the case incidence is high. During periods of low case incidence however, this assumption is no longer appropriate. Therefore, to be conservative and rather err on the side of too large uncertainty intervals, we also consider the credible interval of $R_{e}$ which is obtained by taking the 0.025 and 0.975 quantiles from the posterior distribution of $R_{e}$ using EpiEstim based on the original data $D_t$. The final reported interval is then the union of the credible interval and the 95% bootstrap confidence interval.

### Appendix B: combining types of observations

In real life outbreaks, more than one observation event can originate from a single infection event. For instance, for a diseased patient who eventually dies after having been admitted in the hospital due to an infection, a single infection event can give rise to a number of successive observations such as: a case confirmation event, a record of hospital admission, of ICU admission, and of death. In total in this example, a single infection event gave rise to four delayed observations.

In the framework estimateR adopts, different types of observation events cannot in general be combined into the estimation of a single $R_e$ value [18]. If four types of observations are made, as in the example above, we would recommend independently estimating $R_e$ from each type of observation assuming that the delay distribution specific to each type of event is known. This recommendation is made because each type of observation event is associated with its own (different) inherent sources of biases and its own subgroup in the infected population, with smaller or larger overlaps [18].

Let us consider a specific context, with similarities to the context of data gathering of several countries during the COVID-19 pandemic. For simplicity, we ignore all hospital- and death-related observation events: we assume that the entire fraction of infection events which ends up being recorded is observed via a case confirmation event. Also, we assume that all confirmed cases are symptomatic. Moreover, when infected individuals are tested positive to the infection of interest, they are asked to report the date at which their symptoms started (the symptom onset date). For various reasons, not all positively-tested individuals report this data. We assume the data is collected into a line list of all confirmed cases, with optional symptom onset date attached.

One could treat the confirmed cases and the symptom onset dates as two different observation types, yielding two distinct $R_e$ estimates. However, in this example, symptom onset observation events represent only a subset of all confirmed cases and we have no reason to believe that symptom onset observations do not carry all reporting biases associated with confirmed cases plus other biases specific to their own reporting. Thus, we attempt to make use of the information on symptom onset events differently.

We assume that the delay from infection to case confirmation can be broken down into two independent successive delays: a first delay from infection to symptom onset (the incubation period) and a subsequent delay from onset of symptoms to case confirmation. Symptom onset events can be seen as intermediary steps between infections and case confirmations. As the random delay associated with each observation event is similar to a blurring effect, symptom onset observation events provide a less-blurred image of the original infection events than the case confirmations do. Thus, if the symptom onset date of an individual is known, their date of infection can be better pinpointed than if only their case confirmation date is known. The better the infection events are reconstructed, the better the outbreak dynamics can be reconstructed and the more accurate the $R_e$ estimates.

Thus, when an observation event is an intermediary step on the path to a final observation event, it is desirable to use the former event as the starting point to the infection event reconstruction instead of the latter. estimateR allows to do so by combining the incidence of these two types of events: the intermediary events (we call them “partially-delayed observations”) and the final observation event (we call them “fully-delayed observations”). Symptom onset events and case confirmations as described in the above lines are examples of a pair of partially-delayed and fully-delayed observation events.

When partially-delayed observations are independent from their corresponding fully-delayed observations, i.e. they are not contingent on the corresponding fully-delayed observations, it is straightforward to combine the two types of observations to estimate $R_e$. One simply needs to treat them as two different observation time series, from which to independently infer infection events. The two resulting time series of infection events can then be summed up to build a single time series, from which $R_e$ can be estimated. The only caveat is that there must be no overlap between the two types of observations: each infection event should be recorded as either a partially-delayed or a fully-delayed observation.

In many cases, however, a partially-delayed observation is not independent from, but contingent on, its corresponding fully-delayed observation being observed. In that case, when combining the two types of observations, one needs to account for the fact that each partially-delayed observation is only known once a fully-delayed observation of the same infection event is made. This is precisely the case in the example described above: symptom onset dates are only known once a symptomatic individual is tested positively; symptom onset dates are only known retrospectively, and contingent on a case confirmation. Therefore, recordings of symptom events for time steps close to the present represent only a fraction of the eventual recordings made for these time steps (once all corresponding case confirmations have been made). Thus, the incidence of symptom onsets (and of all partially-delayed observations with similar properties) close to the present underestimates the real incidence and it must be transformed to correct for this effect. A so-called nowcasting procedure is applied to such partially-delayed observations, this procedure accounts for yet-to-be-recorded events: partially-delayed events that have already happened, but have not yet been recorded. To do so, we compute the maximum-likelihood estimator of the eventual number of partially-delayed observations for a particular time step by dividing the number of observations made so far by the probability of such an observation to have been recorded before present [30, 31]. As in the case where partially-delayed are independent from fully-delayed observations, the nowcast partially-delayed observation incidence and fully-delayed incidence can be then be used to independently reconstruct latent infection events, and the two resulting time series of infection events can be summed up into a single series. Again, there must no be any overlap in recorded cases between the partially-delayed and fully-delayed observations.

### Appendix C: simulation procedure

We simulate observations using the following procedure.

#### Simulating infection events

An $R_e$ trajectory is first constructed over 150 time steps, each trajectory translating one of the five scenarios of interest. For each scenario, we simulate 100 outbreaks. Each outbreak is seeded with one imported case per time step for five consecutive time steps. The number of infection events on day *t*, $I_t$, is drawn from a Poisson distribution with mean $R_e(t)\Lambda _{t}$, with $\Lambda _{t}$ as defined in Eq. 9. For the infectivity profile $w_s$, we use the discretised serial interval for SARS-CoV-2: a draw from a Gamma(shape =2.73, scale=1.39) + 1 [32].

#### Generating delayed observations

*Basic validation* Observations are derived from the simulated infections by convolving the infection incidence with a delay distribution, representing the distribution of delays from infection to observation. In the basic validation set up, the delay distribution is the result of the convolution of two delay distributions: a Gamma(shape=3.2, scale=2.1) distribution which could represent an incubation period, and a Gamma(shape=2.7, scale=2.6) distribution which could represent a delay from symptom onset to case confirmation (or hospital admission, or any other type of observation).

*Validation on simulated data generated with time-varying delay distributions* When generating observations with time-varying delay distributions, the delay distribution with which the infection incidence is convolved gradually moves from a shorter delay distribution to a longer one, or vice-versa. This change happens regularly from the start of the simulated outbreak to the simulated present time. Delays are composed of a Gamma (shape = 3.2, scale = 2.1) distribution for the initial incubation period, and a distribution for the delay between onset of symptoms to case confirmation (short delay: Gamma (shape = 2, scale = 2); long delay: Gamma (shape = 2, scale = 8).

*Validation on simulated data containing partially-delayed observations* We generate pairs of partially-delayed and fully-delayed observation series with a slightly different procedure. First, a partially-delayed observation event is generated for each infection event, drawing a sample from a gamma-distribution meant to represent an incubation period Gamma (shape = 3.2, scale = 2.1). Then, from each partially-delayed observation, we simulate a fully-delayed observation event by drawing a sample from a delay distribution representing a delay from symptom onset to case confirmation Gamma (shape = 3, scale = 5). Partially-delayed observations are assumed to be contingent on their associated fully-delayed observation. Thus, we discard partially-delayed observation events with a fully-delayed observation event posterior to the simulated present time, as those partially-delayed observation have not been recorded yet.

We then build two incidence series, the first one for partially-delayed observations and the second for fully-delayed observations. For each infection event, we record the partially-delayed observation event with a probability *p* in the first incidence series. Otherwise, we record the fully-delayed observation event in the second incidence series.

#### Including additional observation noise

To increase the realism of the generated observations [18], we combine them with autocorrelated noise. This noise $\nu _t$ is generated using an autoregressive noise model of order 4 (AR(4)), with coefficients $(ar_1 = 0.05, ar_2 = 0.05, ar_3 = -0.02, ar_4 = -0.02)$ and standard deviation 0.05. Coefficients are selected to loosely approximate country-level empirical COVID-19 incidence data. The number of observations made on time step *t*, with noise, $O_{t}$ is computed from the generated observations $D_t$ with:

12 $$\begin{aligned} O_{t}&= D_{t} \times e^{\nu _t}. \end{aligned}$$

When comparing estimateR to similar existing methods, we use a different type of noise, as we did not manage to obtain meaningful estimates with epidemia and EpiNow2 with the autocorrelated noise. In this case, the noise factor for each time step *t* ($\nu _t$) is an independent random draw from a normal distribution with mean 0 and standard deviation 0.1.

### Appendix D: default settings

In estimateR, by default, the most recent $R_e$ estimate produced corresponds to the time step $N-\mu$, with *N* being the most recent available time step and $\mu$ being the median of the delay distribution. This truncation is done as posterior $R_e$ estimates are too uncertain. When dealing with a combination of partially and fully delayed data, the default setting is slightly more complex. In this case, the most recent $R_e$ estimate corresponds to the time step $(N-Y) - \mu$ with *Y* being the 33rd percentile of the delay distribution between partially-delayed and fully-delayed observation, *N* and $\mu$ carry the same meaning as previously. In other words, we first exclude the *Y* most recent time steps for which a partially-delayed observation has a probability less than 0.33 to be fully observed before the most recent time step. The default threshold of 0.33 was chosen as a trade-off between certainty in the result and timeliness of the most recent $R_e$ estimate.

### Appendix E: parameterization for dengue fever

We used line list data of cases with dengue fever in Rio de Janeiro, Brazil, to fit a parametric distribution for the delay between the date of symptom onset and the date of recording in the Brazilian Information System for Notifiable Diseases [23]. Since we found no substantial change in reporting delay during the relevant time period, we fitted the same delay distribution for the full time horizon, yielding a log-normal distribution with parameters $\mu =2.90$ and $\sigma =0.83$. For the intrinsic (i.e. human) incubation period, we used a log-normal distribution with a mean 5.90 days and standard deviation of 1.60 days [21]. In the case of dengue fever, generation intervals are likely to be temperature-dependent [22]. For simplicity, we here used a generation interval for the transmission from human to human via a mosquito vector that is in line with the average annual temperature in Rio de Janeiro ($27.2^\circ C$), although we note that in practice, higher accuracy may be obtained by using time-varying intervals [33]. Specifically, we used a gamma distribution with a mean of 23 days and a standard deviation of 8.5 days [22]. Due to the long generation interval and delays, we chose the LOESS smoothing parameter $\sigma$ in estimateR such that a broad time window of 10 weeks is covered. We used EpiNow2 with default settings and a day-of-the week effect for the reported cases.

## Abbreviations

AR(*n*): Autoregressive model of order *n*
COVID-19: Coronavirus disease 2019
LOESS: Locally estimated scatterplot smoothing
*R*_e_: Effective reproductive number
RMSE: Root mean square error
SARS-CoV-2: Severe acute respiratory syndrome coronavirus 2
