# Supplementary figures and images for: estimateR: an R package to estimate and monitor the effective reproductive number
Source: BMC Bioinformatics. 2023 Aug 11;24:310. doi: 10.1186/s12859-023-05428-4 (PMC10416499; doi:10.1186/s12859-023-05428-4)

(A) Reproductive number

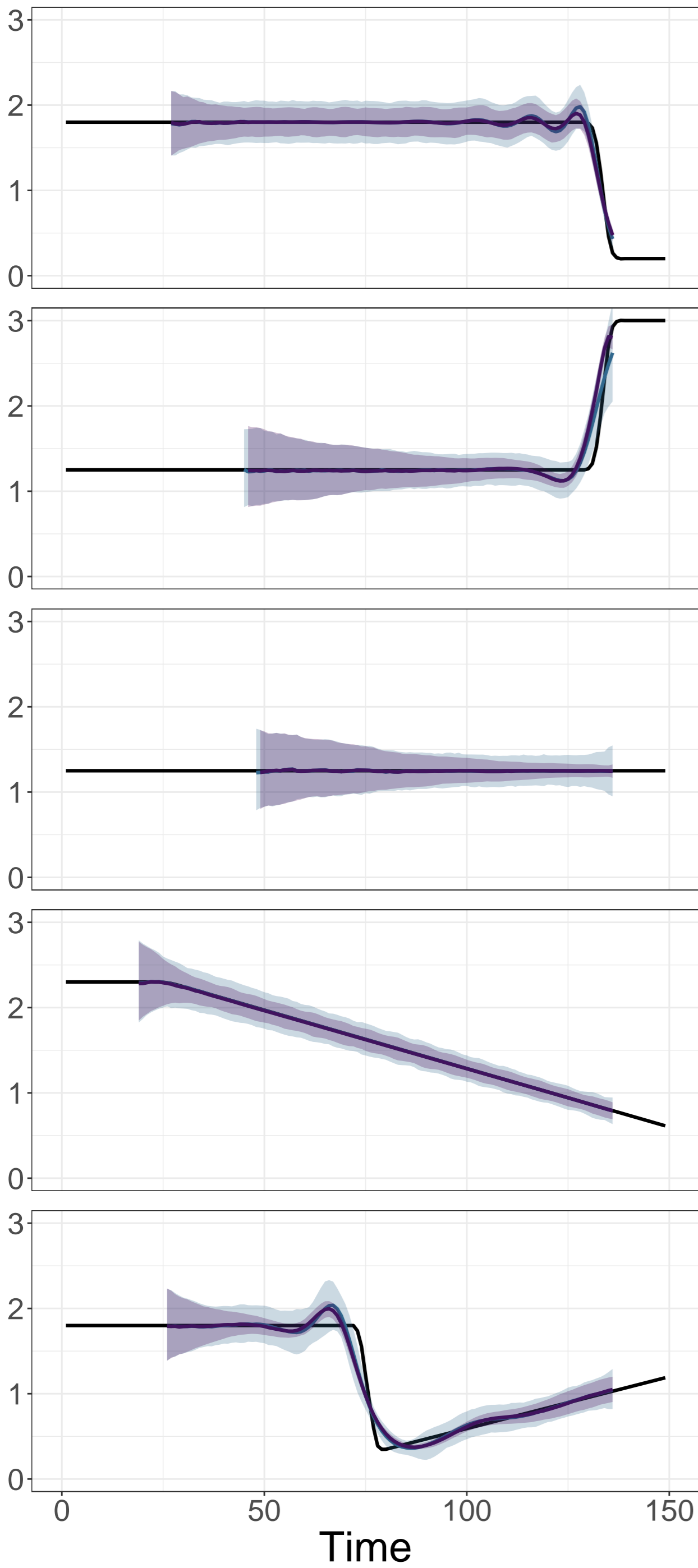

(B) Coverage

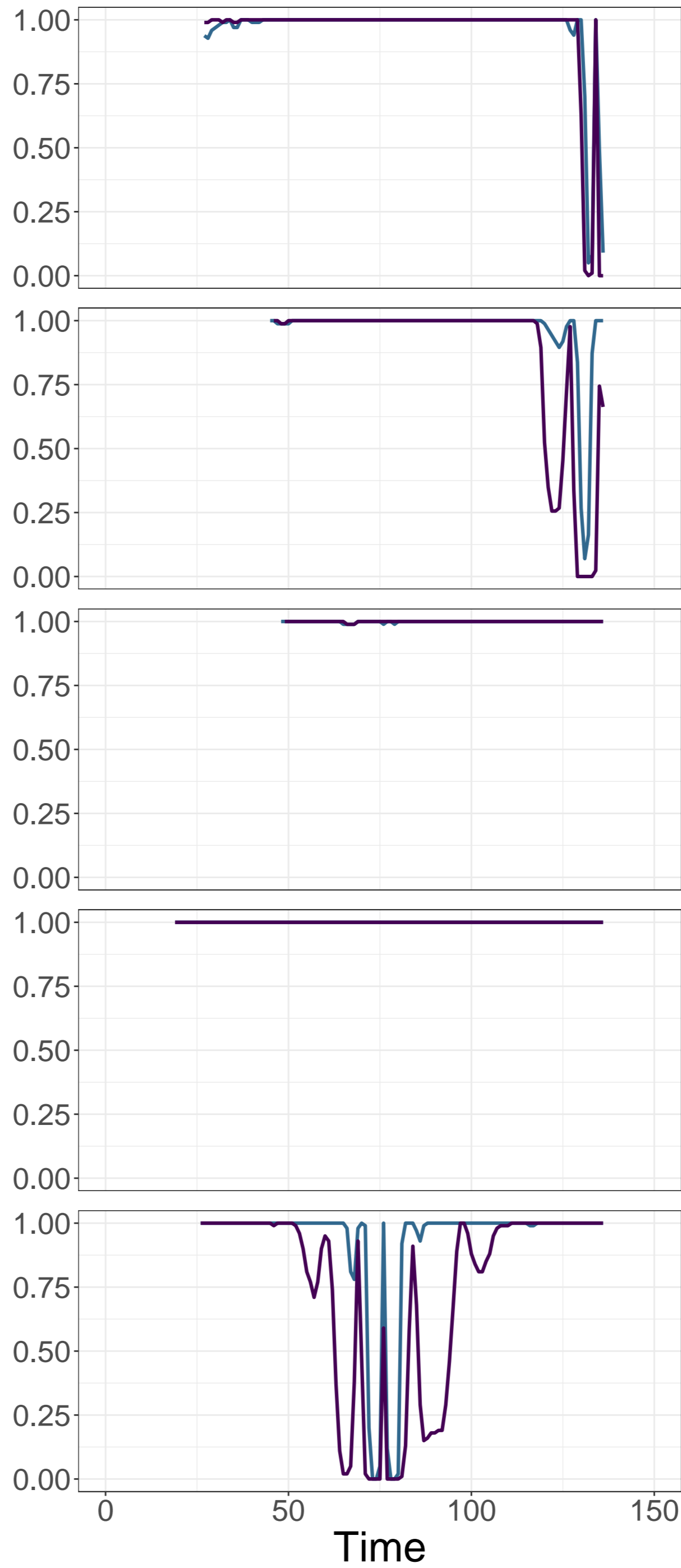

(C) RMSE

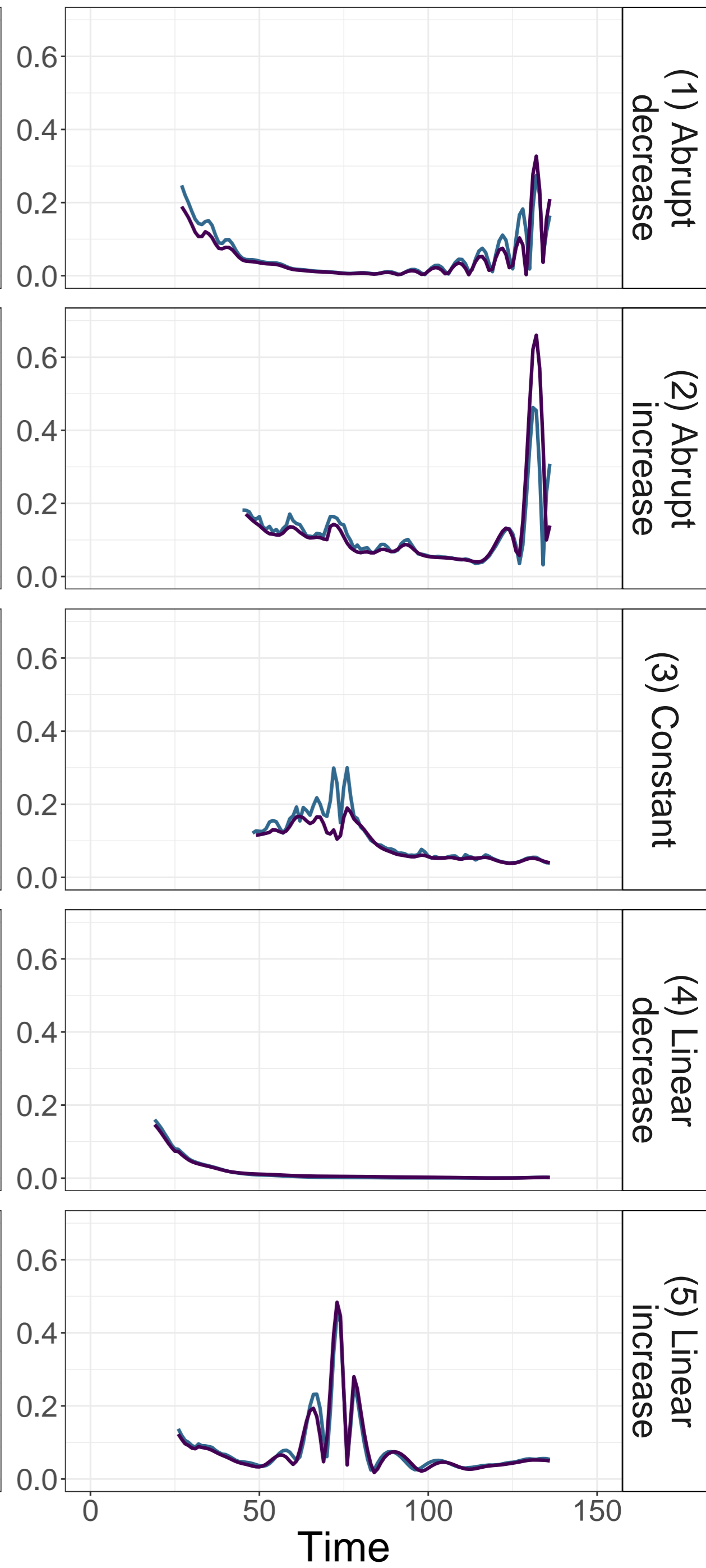

Smoothing: No smoothing Smoothing

Supplement: Supplementary file 1 — Additional file 1: Fig. S1. Summary of Re inference on simulated data without added observation noise, obtained with and without an initial smoothing step. Each row corresponds to a different scenario of Re changes through time. The first column shows the ground truth as a black line, and the median (lines) and lower and upper bounds of the 95% confidence interval (ribbons) of Re estimates obtained over 100 replicates, with (purple) and without (blue) an initial smoothing step, respectively. The second column shows corresponding coverage values (fraction of replicates for which the ground truth is inside the confidence intervals) and the third column shows root mean squared error (RMSE) values for each scenario. [file 12859_2023_5428_MOESM1_ESM.pdf]

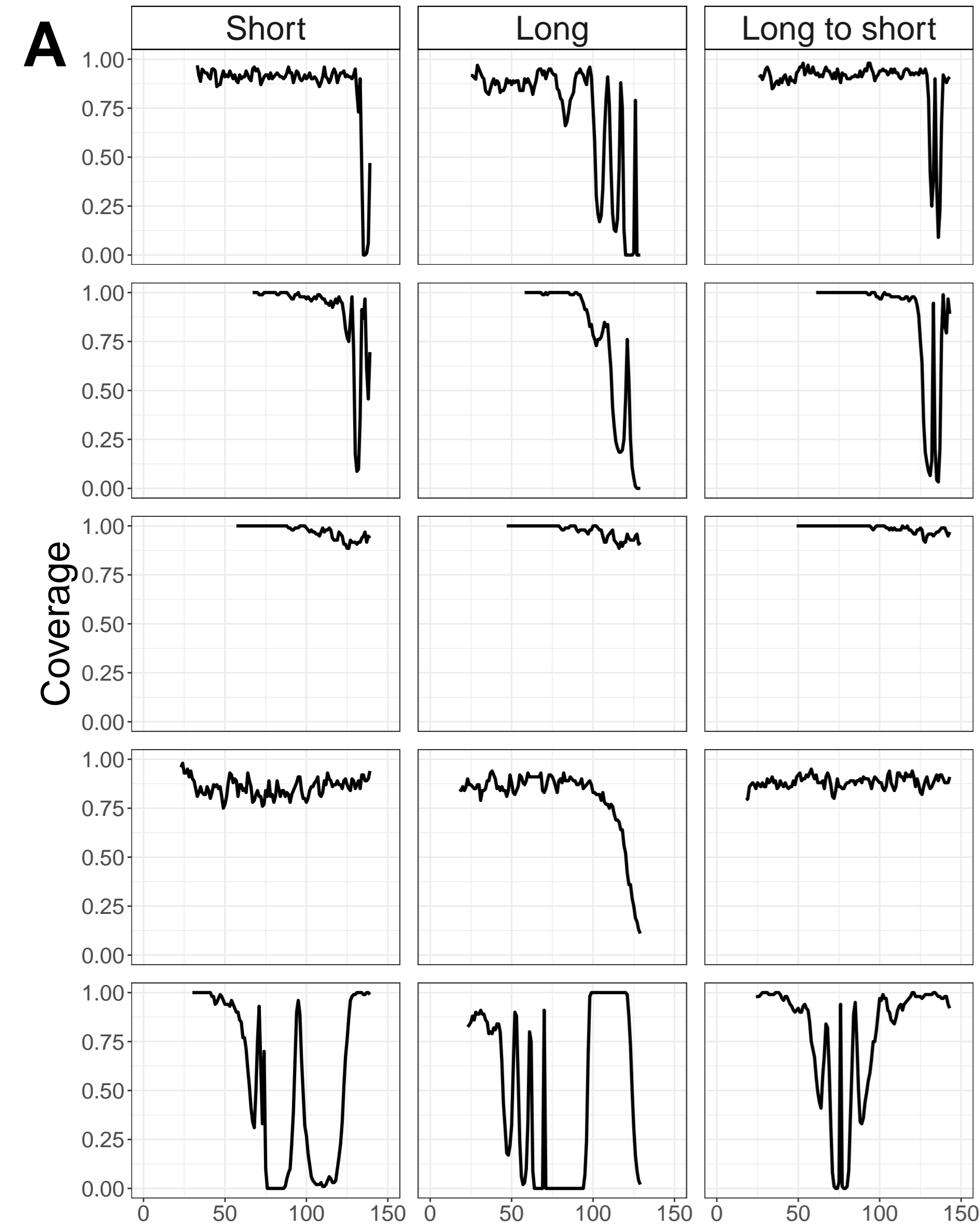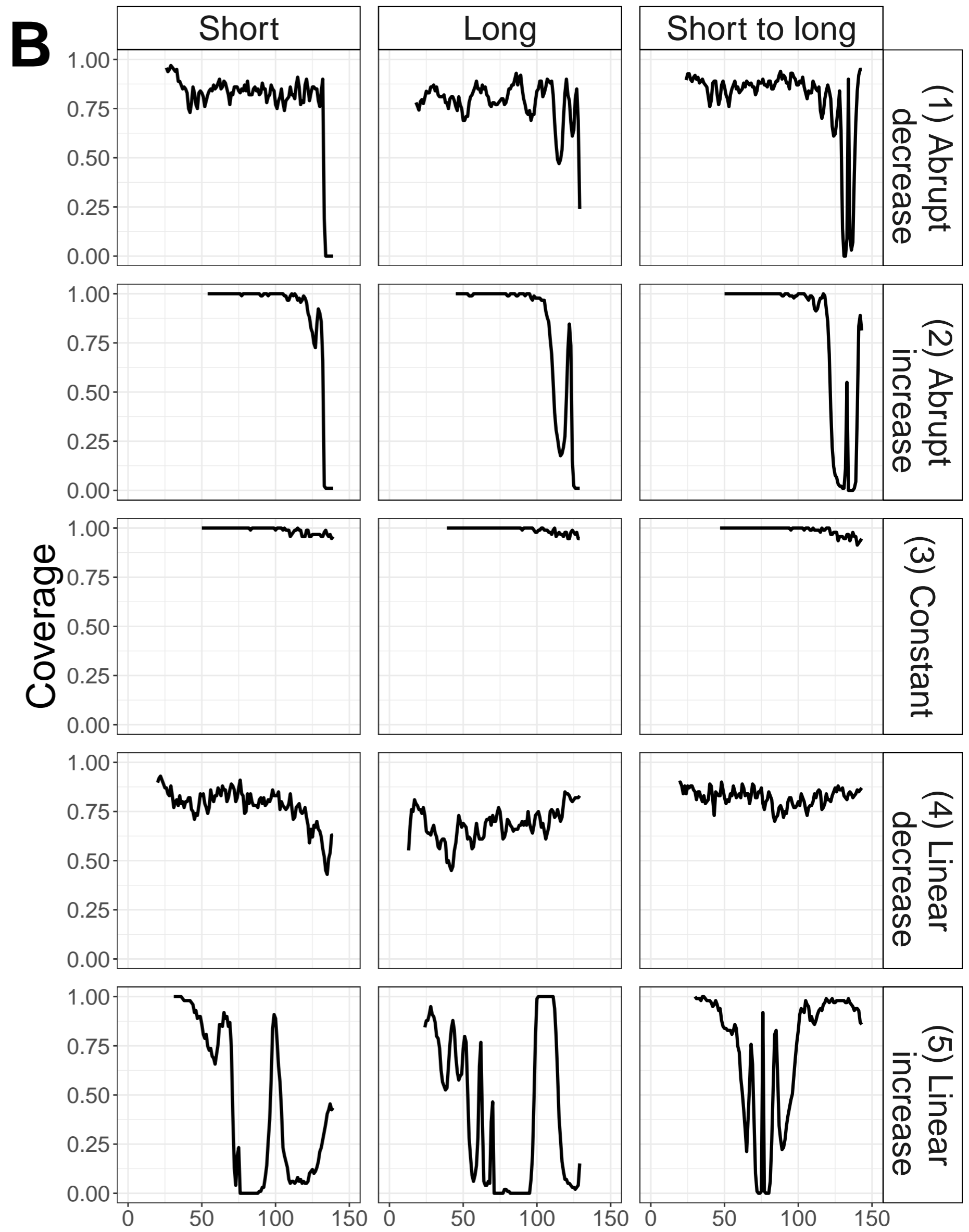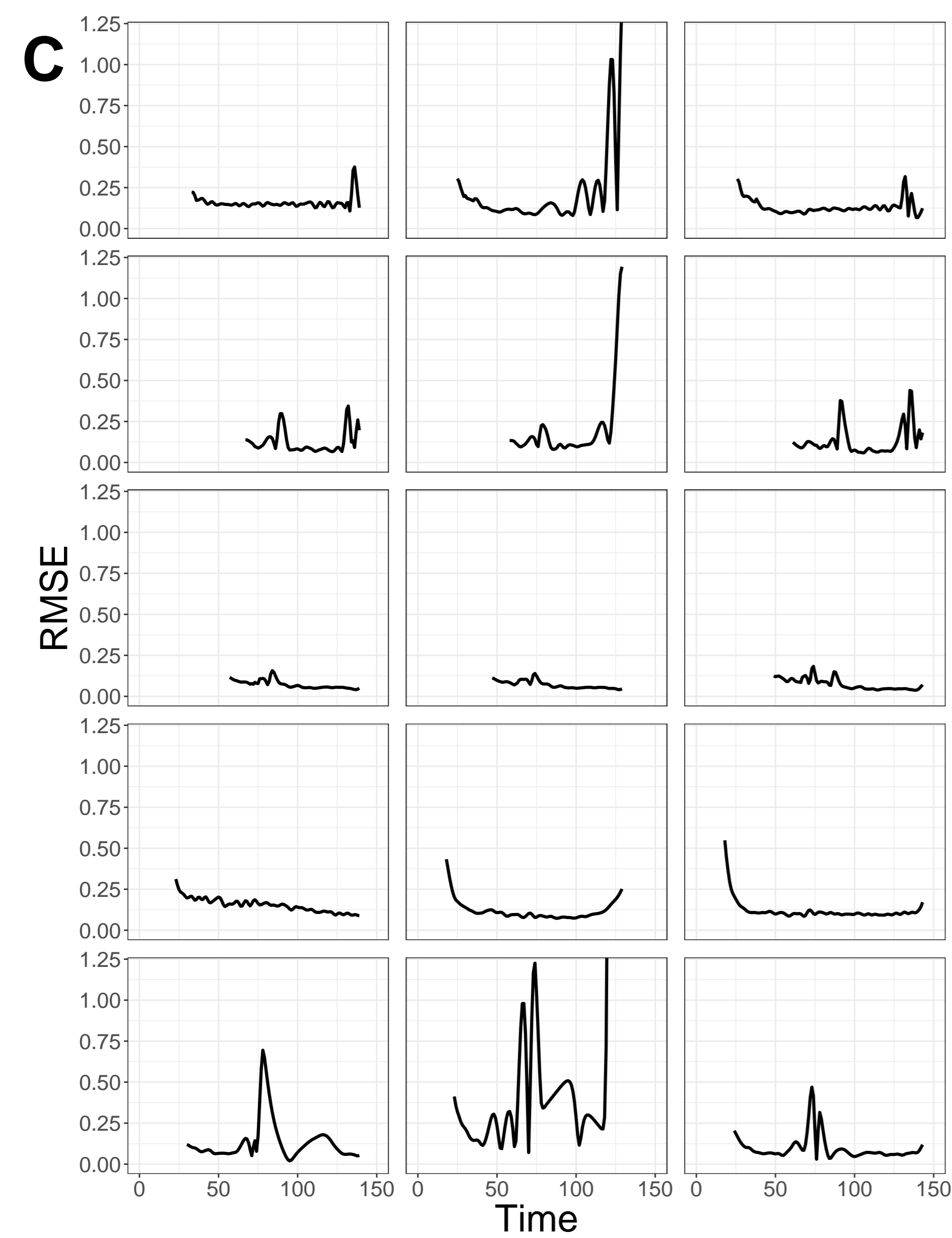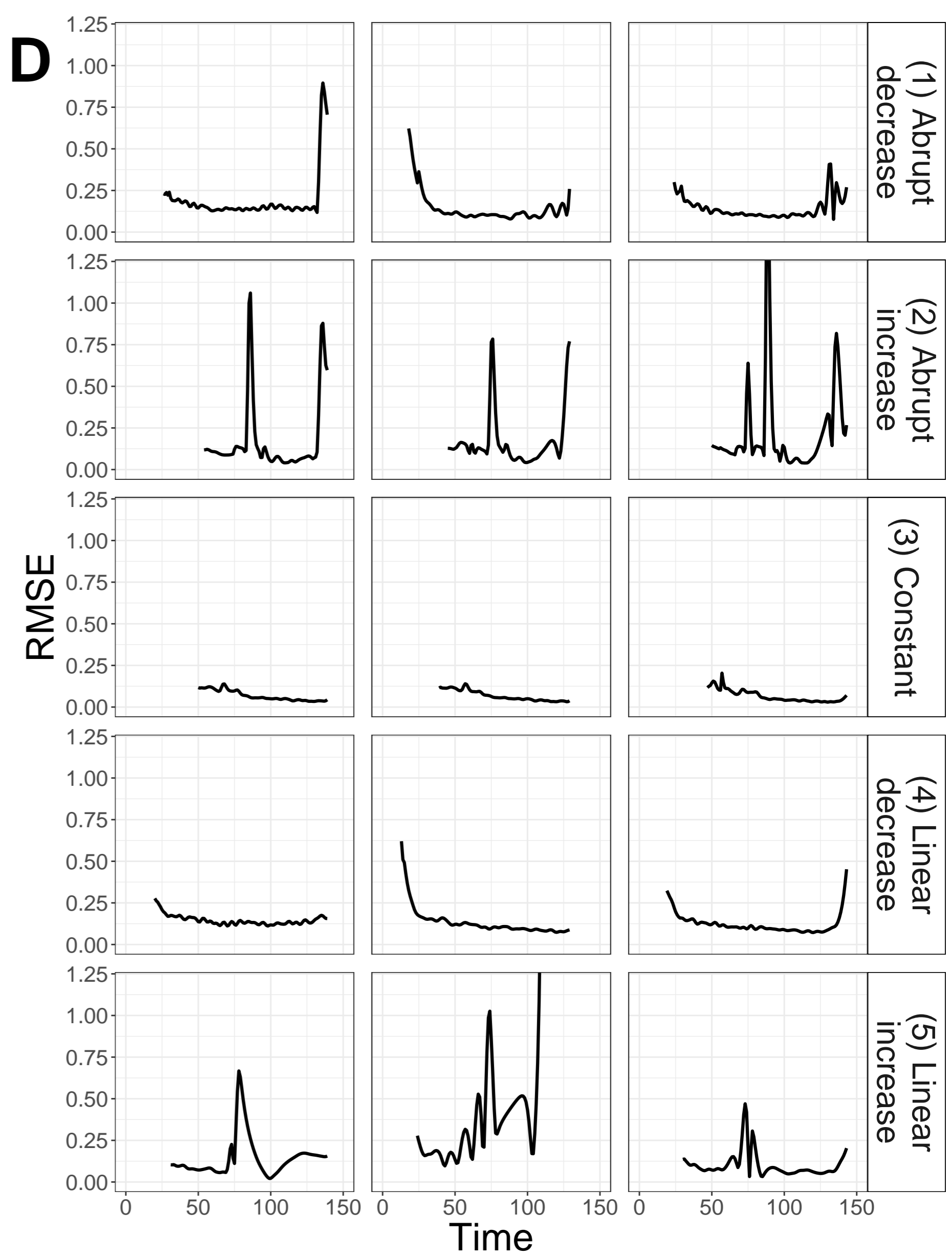

Supplement: Supplementary file 2 — Additional file 2: Fig. S2. Coverage and RMSE values on Re estimates on simulated data with time-varying delay distributions. Each row corresponds to one of five Re scenarios. Each column corresponds to a different delay distribution in the analysis. In the first two columns, delay distributions are fixed and either short or long. In the third column, delay distributions are allowed to vary when estimating(from short to long or long to short). A and C: Coverage and RMSE values on Re estimates on simulated data with observation delays gradually changing from a long (at time 0) to a short (at time 150) observation delay distribution. B and D: Coverage and RMSE values on Re estimates on simulated data, with observation delays gradually changing from a short (at time 0) to a long (at time 150) observation delay distribution. [file 12859_2023_5428_MOESM2_ESM.pdf]

**A**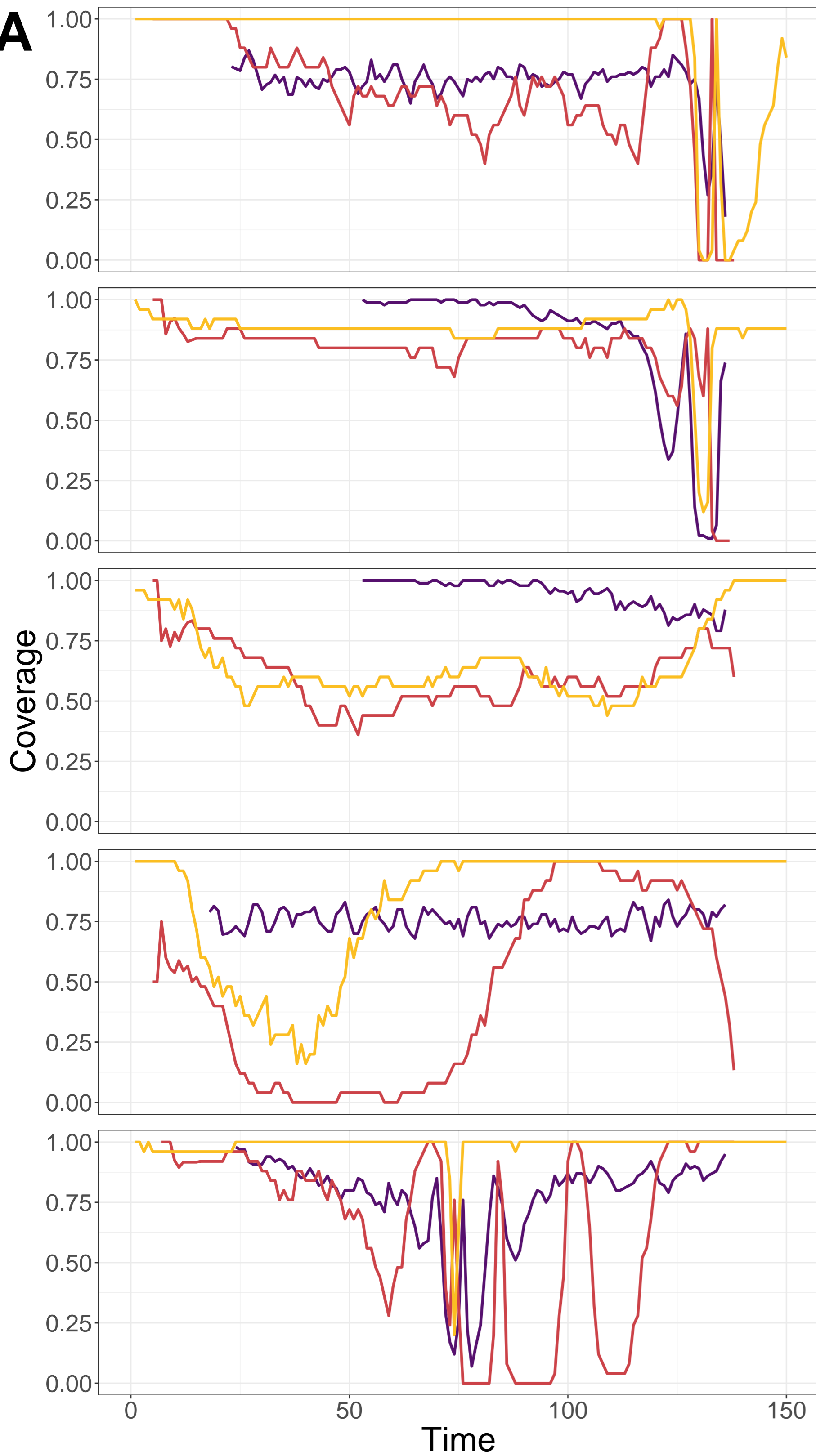**B**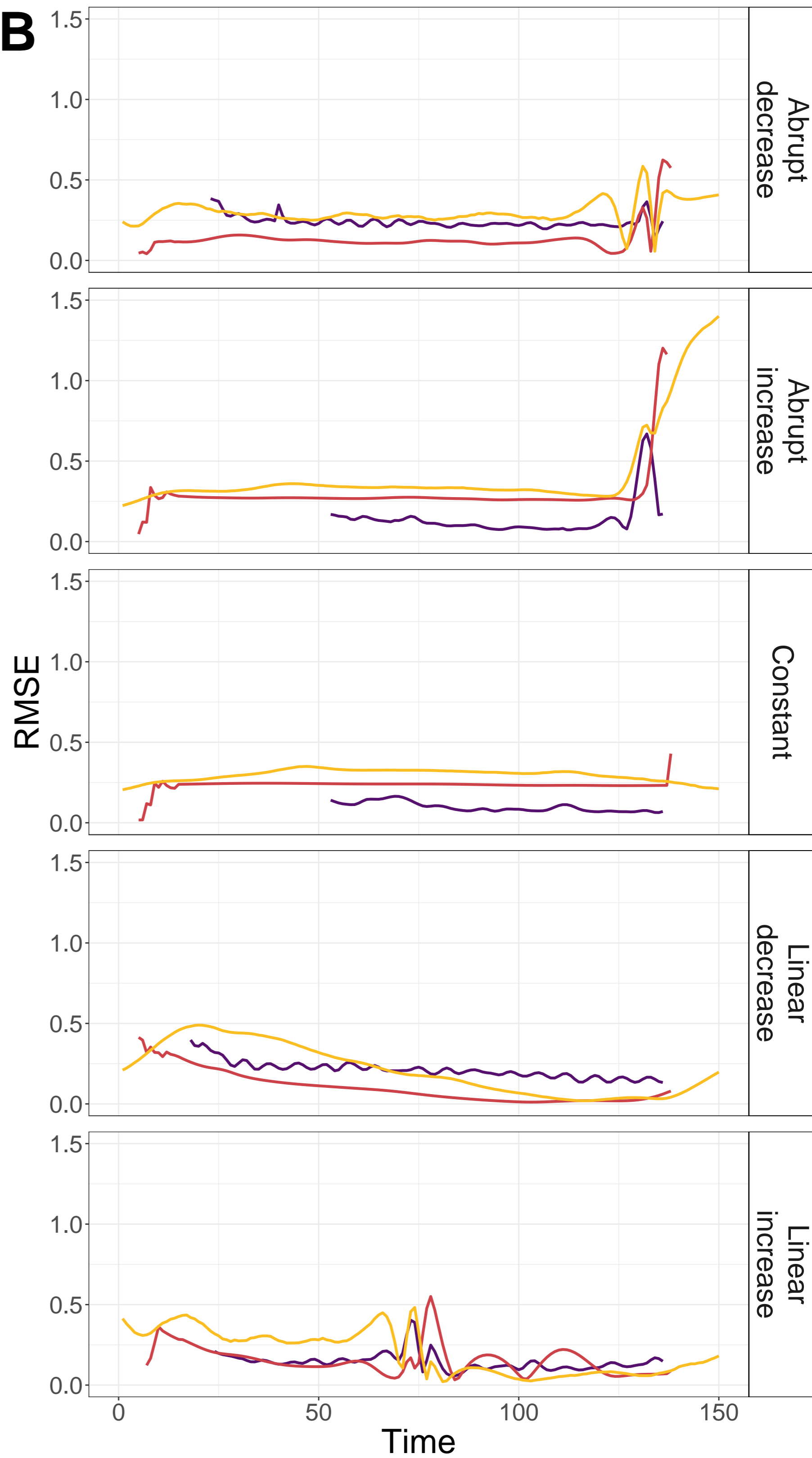

R package: — estimateR — EpiNow2 — epidemia

Supplement: Supplementary file 3 — Additional file 3: Fig. S3. Coverage and Root Mean Squared Error of Re estimates using estimateR, epidemia and EpiNow2. The rows show five scenarios of Re variations through time. A: Coverage values (fraction of replicates for which the ground truth is inside the confidence intervals). B: Root Mean Squared Error (RMSE) values. [file 12859_2023_5428_MOESM3_ESM.pdf]
